# MegaSSR: a web server for large scale microsatellite identification, classification, and marker development

**DOI:** 10.3389/fpls.2023.1219055

**Published:** 2023-12-14

**Authors:** Morad M. Mokhtar, Alsamman M. Alsamman, Achraf El Allali

**Affiliations:** ^1^ Bioinformatics Laboratory, College of Computing, Mohammed VI Polytechnic University, Benguerir, Morocco; ^2^ Agricultural Genetic Engineering Research Institute, Agricultural Research Center, Giza, Egypt; ^3^ Biotechnology Department, International Center for Agricultural Research in the Dry Areas (ICARDA), Giza, Egypt

**Keywords:** Simple Sequence Repeats, bioinformatic pipeline, MegaSSR web server, SSR markers, Non-redundant SSR library

## Abstract

Next-generation sequencing technologies have opened new avenues for using genomic data to study and develop molecular markers and improve genetic resources. Simple Sequence Repeats (SSRs) as genetic markers are increasingly used in molecular diversity and molecular breeding programs that require bioinformatics pipelines to analyze the large amounts of data. Therefore, there is an ongoing need for online tools that provide computational resources with minimal effort and maximum efficiency, including automated development of SSR markers. These tools should be flexible, customizable, and able to handle the ever-increasing amount of genomic data. Here we introduce MegaSSR (https://bioinformatics.um6p.ma/MegaSSR), a web server and a standalone pipeline that enables the design of SSR markers in any target genome. MegaSSR allows users to design targeted PCR-based primers for their selected SSR repeats and includes multiple tools that initiate computational pipelines for SSR mining, classification, comparisons, PCR primer design, *in silico* PCR validation, and statistical visualization. MegaSSR results can be accessed, searched, downloaded, and visualized with user-friendly web-based tools. These tools provide graphs and tables showing various aspects of SSR markers and corresponding PCR primers. MegaSSR will accelerate ongoing research in plant species and assist breeding programs in their efforts to improve current genomic resources.

## 1 Introduction

Microsatellites are a class of DNA repeats that include Simple Sequence Repeats (SSRs), repeats of 1 to 6 bp distributed throughout the eukaryotic genome (Phumichai et al., 2015). These repeats may be distributed throughout the genome with or without short interruptions and may cover a substantial portion (*>* 50%) of the genome (Haubold and Wiehe, 2006). Because of advances in DNA sequencing technology over the past decade, numerous genomic and transcriptomic datasets have been published. Researchers have used this information to investigate the abundance and impact of SSR motifs on the functionality and structure of animal and plant genomes (Mokhtar and Atia, 2019). Several studies suggest that SSRs are not randomly distributed across the genome (Vieira et al., 2016).

SSRs are subject to random genetic mutation at higher rates than other parts of the genome, with long SSR motifs having a higher mutation rate than short SSR motifs (Vieira et al., 2016). Due to errors in DNA replication or the recombination process, genetic mutation results in the addition or deletion of SSR motifs. New SSR alleles can be formed due to errors in the DNA mismatch repair system. They lead to the formation of different SSR alleles, and these polymorphisms are passed on to the next generation (Vieira et al., 2016). Because of their significant contribution to genetic variation, SSRs have attracted the interest of molecular evolutionary researchers. SSRs have been used as codominant, multiallelic, repeatable, highly informative, and transferable PCR-based markers to study related and distant species (Mason, 2015). Over the past decade, SSR markers have been used in a variety of evolutionary studies, for genotyping, diversity, marker-assisted selection, linkage map construction, integrated maps, physical and sequence-based maps, and quantitative traits loci (Garcia et al., 2006; Kalia et al., 2011; Souza et al., 2013; Hayward et al., 2015).

SSR research continues to expand due to its undeniable importance in genome assembly, annotation, and gene regulation. For decades, SSR markers have been successfully used to select potential varieties for breeding programs, and several studies have linked microsatellite instability to phenotypic variability (Li et al., 2004; Gao et al., 2013). This link has made SSR an important tool for breeders and geneticists to study genetic variation in relation to phenotypic variation in organisms (Hayward et al., 2015). According to the PubMed and Scopus search engines, SSRs have been used in thousands of research articles in recent years to study molecular ecology, conservation biology, phylogenetic diversity, genetic markers for breeding, and many other areas. The identification of SSR motifs has become increasingly important in recent years, and several computational algorithms have been developed to detect their occurrence in the genomic sequence. The utility of these tools is primarily determined by their ability to identify complex SSR structures, their flexibility, their ease of maintenance, and the minimal computer skills required for proper use. These tools include TRF (Benson, 1999), TROLL (Castelo et al., 2002), mreps (Kolpakov et al., 2003), SciRoko (Kofler et al., 2007), MsDetector (Girgis and Sheetlin, 2013), GMATo (Wang et al., 2013), GMATA (Wang and Wang, 2016), MISA (Thiel et al., 2003), PolyMorphPredict (Das et al., 2019), ESAP Plus (Ponyared et al., 2016), SAT (Dereeper et al., 2007), AARTI (Kumar et al., 2022), ESMP (Sarmah et al., 2012), WebSat (Martins et al., 2009), SSRPrimer (Jewell et al., 2006), WGSSAT (Pandey et al., 2018), and IMEx (Mudunuri and Nagarajaram, 2007). Among these tools, MISA is a widely used tool for SSR detection due to its early development, efficiency, and simplicity.

The expansion of genomic sequencing data requires the development of simple platforms for SSR detection, classification, and comparison. Currently available tools for SSR identification have one or more major limitations that hinder their adoption on a larger scale. Several of these tools have limited ability to examine large genomic datasets, do not use publicly available gene annotation data, do not have graphical interfaces that allow manipulation of results, or do not provide tools for genome-wide analyses and assessments. Although some of these tools, such as the GMATA pipeline (Wang and Wang, 2016), have attempted to avoid most of these limitations, it still has some drawbacks, such as the lack of classification and comparison of SSR motifs based on their genomic location and the lack of an online version. The availability of an online version of SSR detection tools should facilitate the current and future inclusion of SSR markers in basic and advanced research studies.

Here, we developed MegaSSR as a web server for large-scale SSR identification, classification, and marker development. The proposed online pipeline provides a wide range of useful and routine tools for automatic and easy identification, classification and annotation of SSR markers. This pipeline is supported by the fastest supercomputer in Africa. MegaSSR provides a centralized framework for the study, manipulation, and design of targeted PCR-based SSR markers at the whole genome and transcriptome level. The key steps in the MegaSSR pipeline are: 1) SSR mining; 2) SSR classification; 3) SSR gene-based annotation; 4) SSR motif comparison; 5) SSR primer design; and 6) statistical visualization. MegaSSR is a unique and useful tool for filtering SSRs and PCR-based primers based on genomic location and proximity to functional genomic regions. It is also available as a standalone program that can be easily installed in the Conda environment.

## 2 Materials and methods

The computational pipeline of the MegaSSR web server and its data resources consists of various subsystems interconnected by data adapters. These adapters ensure that data is passed from FASTA sequences to processed data and statistics in an end-to-end pipeline.

### 2.1 Implementation

**Figure 1:**
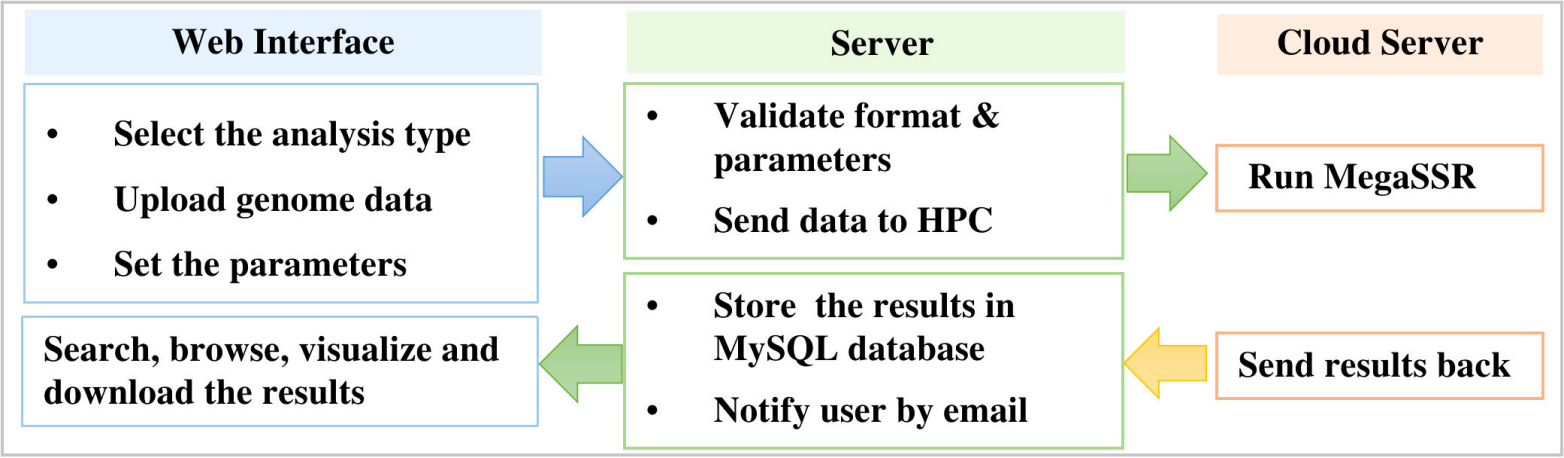
Workflow to manage submitted jobs and processes between the web server and the high performance computer.

MegaSSR is hosted on LAMP server: Linux 5.4.0-89-generic x86 64 (Ubuntu 20.04.3 LTS), Apache (version 2.4.41), MySQL (version 8.0.27), and PHP (version 7.4.3). Perl (v5.30.0), Python (v3.8.10), R (v4.1.2) are installed as a prerequisite for the software and tools used in the computational pipeline. The LAMP server runs on a computer with 32GB of memory, 16-core CPUs and a 10TB hard drive. HTCondor (v9.5.0) is used to manage and schedule the submitted tasks and processes. After the server validates the uploaded data format, the jobs are sent to the fastest high-performance computer in Africa (TOUBKAL-POWEREDGE C6420, CRC-STACKHPC, XEON PLATNIUM 8276L 28C 2.2GHZ, MELLANOX INFINIBAND HDR100. https://www.top500.org/system/179908/) and the results are sent back to the web server (**Figure 1**).

**Figure 2:**
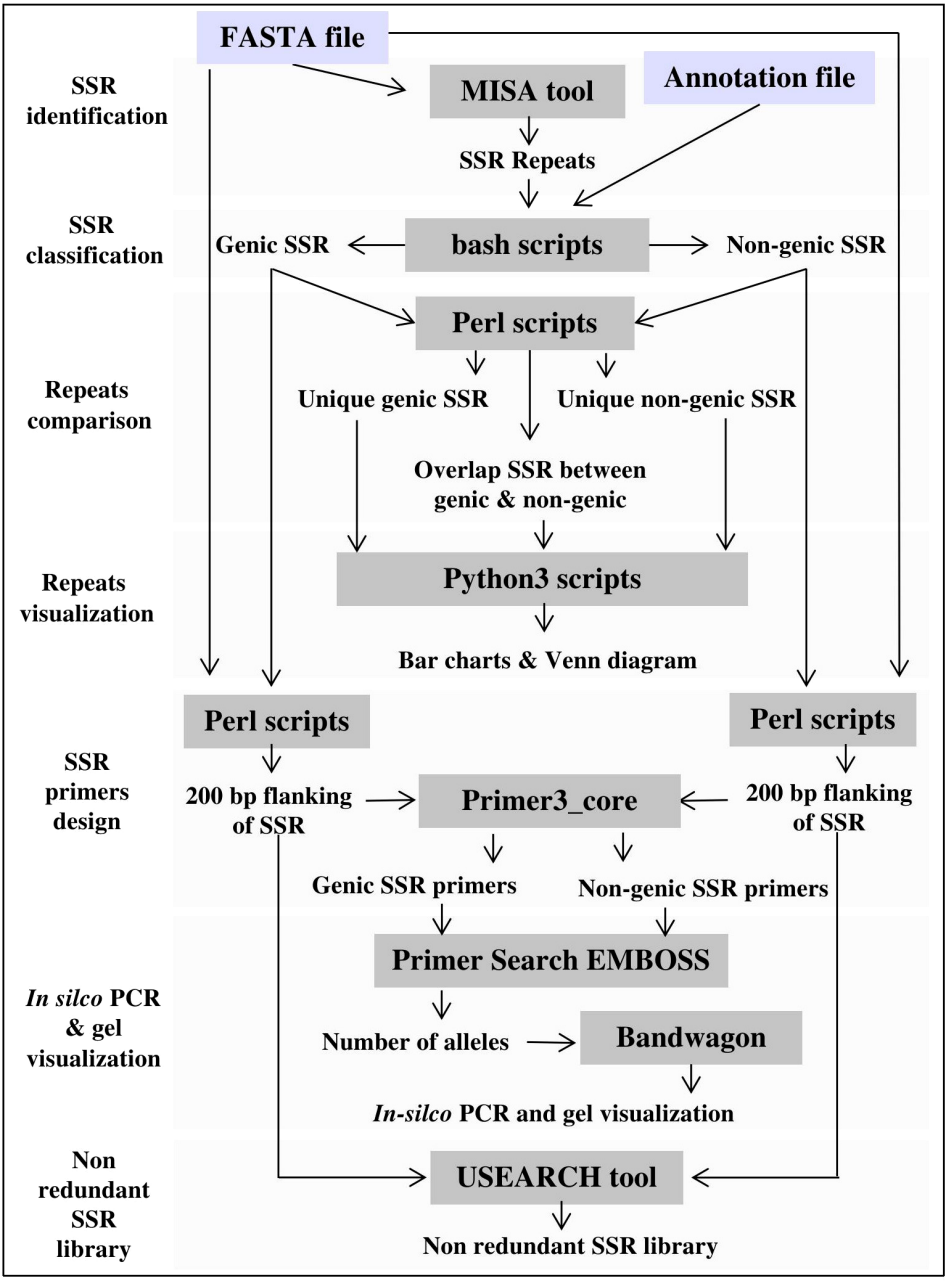
MegaSSR framework, including pipelines for SSR mining, statistical analysis and results visualization.

The computational pipeline consists of the Microsatellite Identification Tool (MISA v1.0) (Thiel et al., 2003), Primer3 PCR primer design software (v2.6.1), and Primersearch (EMBOSS v6.6.0) for *in silico* validation of designed SSR primers. Other processes, such as SSR classification, annotation, and motif comparisons, are performed using custom scripts written in different programming languages. Several software and programming libraries are used to create visualization plots, including Google Charts (https://developers.google.com/chart), ggplot2 v3.3.3 (R package), Bandwagon v0.3.2 (https://github.com/Edinburgh-Genome-Foundry/BandWagon), and the JBrowse v1.0 (Buels et al., 2016). JBrowse is used to map, visualize, and localize SSR motifs and to generate PCR-based primers and their associated annotations at whole-genome scale. Finally, PHP, Cascading Style Sheets (CSS), HTML, and JavaScript were used to create the website interface. **Figure 2** illustrates the computational framework of MegaSSR.

The MegaSSR workflow consists of several steps as shown in **Figure 2**. The pipeline starts with data preparation, where several quality control scripts are used to ensure that the uploaded files are in the correct format. On the main page, the user is notified if data is unreadable or incorrectly formatted. The SSR identification process begins with the submission of data to the MISA (Thiel et al., 2003). The MISA tool is used to identify perfect and compound SSR motifs. Users can change the default parameters to ensure that the submitted analysis is more specific to the data provided. The default parameters are mononucleotide ≥ 10 units, dinucleotide ≥ 6 units, trinucleotide ≥ 5 units, tetranucleotide ≥ 4 units, pentanucleotide ≥ 3 units, and hexanucleotide ≥ 3 units. For compound SSR motifs, the default maximum difference between the two motifs is 100 bp. These default parameters were chosen based on previous SSR studies (Mokhtar et al., 2016; Mokhtar et al., 2020). The generated SSR units go through the steps of classification, assembly, and clustering. After classification into different categories and assembly, the units are clustered based on motif class, genomic position, and gene annotation. The flanking regions of the identified SSR units are extracted from the provided genomic data. These sequences will be used to generate SSR-specific primers for PCR analysis and create a non-redundant SSR library. Primer3 (Untergasser et al., 2012) is used to design SSR-targeted primers based on the user-defined parameters. Users can also use the default parameters, which include primer lengths from 10 to 22 bp, a melting temperature of 55°C, a G/C content of 50%, and a PCR product size range of 100-500 bp. USEARCH v11.0 (Edgar, 2010) is used to create a non-redundant SSR library with a minimum sequence identity of 90%. All data generated by the MegaSSR pipeline are used to calculate a variety of statistical measures for post-processing and to generate tables and graphs. Bang and Chung (2015) reported that there is a risk in using length variation of SSR without sequence confirmation, even within a species. To avoid this risk, MegaSSR provides users with SSR flanking sequences as FASTA files. In addition, MegaSSR reports potentially amplified bands and their length variations within the same genome using *in-silico* PCR. This helps to ensure the accuracy and reliability of the results obtained from MegaSSR. The previous processing steps are completed in sequence. If successful, users are notified via the processing page when the steps are complete, or via email (if one is provided) when the entire analysis is complete. The results generated by MegaSSR can be viewed and downloaded from the website for one month using the link provided, or users can search for them on the homepage using the unique process ID.

### 2.2 Standalone version

MegaSSR is also available as a standalone mode (https://github.com/MoradMMokhtar/MegaSSR). It has been tested on Ubuntu 18.04 and 20.04 and can be installed through the Conda environment with the command “conda env create -f MegaSSR.yml”, which installs all MegaSSR dependencies. In standalone mode, the user can set all parameters, including SSR identification, primer design, *in silico* PCR, and the number of threads to use. The parameters are flags such as the analysis type (*-A*) fasta file (*-F*) GFF file (*-G*) outfile prefix (*-P*) minimum number of mononucleotides (*-1*) dinucleotides (*-2*) trinucleotides (*-3*) tetranucleotides (*-4*) pentanucleotides (*-5*) hexanucleotides (*-6*) maximum difference between the two motifs (*-C*) minimum primer length (*-s*) maximum primer length (*-S*) optimal primer length (*-O*) PCR product size (*-R*) number of CPU/threads (*-t*) calculate the number of alleles for each SSR primer and plot the migration patterns of the DNA bands (*-B*) the maximum allele length (*-L*) number of primers in each image (*-I*).

## 3 Results and discussion

In this section, we provide an overview of MegaSSR’s capabilities using two case studies with whole genomes and transcriptomes. We also compare MegaSSR with other SSR web servers and verify the quality of the identified SSRs using a well-established dataset.

### 3.1 Web server usage

**Figure 3:**
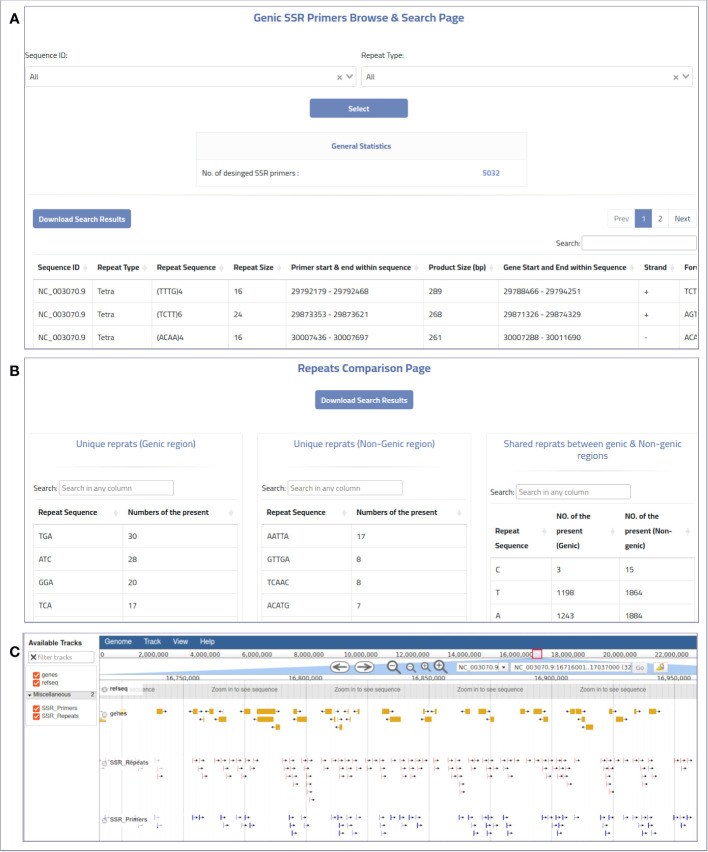
An example of the online MegaSSR output: **(A)** Genic SSR primers browse and search page, **(B)** Repeats comparison page showing a comparison between SSR repeats that are unique to genic and non-genic regions, and shared repeats between them, **(C)** JBrowse visualization.

The web-based interface can be used to provide MegaSSR with the required data. The pipeline accepts two types of input: Fasta sequences and their annotation. Users can upload the whole genome, transcriptome, contigs, ESTs, or any form of nucleotide sequences (FASTA format) from their local computer or via an NCBI-FTP link. In addition, users are encouraged to provide as much information about the target genomic sequences as possible using a general feature format (gff or gff3) file. These features are used to select SSR units near or within genes or any genome features of interest. The web server automatically generates well-designed visualizations that allow users to explore the results and evaluate the SSRs and PCR primers. Users can categorize and select the generated SSR primers based on their functional genomic location and relevance to gene targeting methods or population diversity analyses. The MegaSSR pipeline generates a series of statistical visual representations and tables detailing the statistics of the identified SSR motifs. These results describe, classify, and compare the discovered SSR units based on their distribution in the genomic data, motif class, and proximity to genic regions. MegaSSR generates SSR primers that target the flanking regions of the discovered SSR repeats. The user can filter or classify these primers based on their potential use. The results table displays some important information about the selected forward and reverse PCR primers, such as genomic position, sequence, melting temperatures, and GC content. Some of this information is statistically represented in generated graphs where PCR primers can be classified based on their distance from gene regions (**Figures 3A, B**).

The genome visualization tool JBrowse is used to display various results from the MegaSSR pipeline. These data are presented using genome coordinates. The JBrowse visualization page displays the identified SSR motifs, designed SSR-targeted PCR primers, and gene annotations. Users can explore all relevant information such as genome location, SSR class, SSR sequence length, SSR sequence, primer sequences, and primer product sequence by selecting the coordinate of a specific SSR unit or PCR primer target region. In addition, the JBrowse tool provides an overall view of all SSR units or genes in the genomic regions explored (**Figure 3C**). This information could be helpful in selecting specific SSR units or PCR primers for functional and diversity studies. Users can search and browse the results and also download the results in bulk. The results are provided in the form of tables and figures, as described in **Supplementary Table 1**.

### 3.2 Case study 1: Detection of SSRs at the whole genome level

A number of 35 genome sequences totaling 31.17 giga base pairs from model and non-model organisms were downloaded from the NCBI (Wheeler et al., 2007) and used to validate the performance of MegaSSR in different domains of life. The organisms studied belong to Plantae, Protozoa, Animalia, Chromista, Fungi, Archaea and Bacteria. Accordingly, using the default parameters (implementation subsection), with the exception of mononucleotides, which were excluded from the analysis, a total of 25,339,218 SSR motifs and 7,094,267 SSR primers were detected in the organisms studied. Using 56 CPUs, we report the pipeline execution time for the example genomes in **Table 1**. The genome size of the studied organisms ranges from 4.64 Mb (*Escherichia coli*) to 2866.14 Mb (*Homo sapiens*). The number of pseudomolecules/scaffolds ranges from 1 (*Escherichia coli*) to 16,236 (*Sesamum indicum)*. The run time depends on the genome size and the number of pseudomolecules/scaffolds and ranges from 19 seconds (*Escherichia coli*) to 24 hours (*Anolis carolinensis*). As shown in **Table 1**, MegaSSR is able to analyze the *Oryza sativa* reference genome in 15 minutes. **Supplementary Table 2** provides the organism name, NCBI accession number, organism classification, genome size, total number of SSRs identified, total number of SSR primers developed, and links to download the results for each genome examined.

**Table 1:** Name of organisms, classification, run time (hours: minutes: seconds) using 56 CPU, number of pseudomolecules/scaffolds, genome size (Mbp), number of identified SSRs, and number of designed SSR primers of the validated genomes.

Organism name	Classification	Run time	Pseudomolecules	Genome size	No. of SSRs	No. of SSR primers
*Brassica rapa*	Plant	0:45:13	1,100	352.98	186,389	57,457
*Medicago truncatula*	Plant	0:26:56	42	430.01	332,115	84,590
*Oryza sativa*	Plant	0:14:26	58	374.42	176,760	81,528
*Physcomitrella patens*	Plant	0:31:50	359	472.08	423,673	65,594
*Populus trichocarpa*	Plant	1:31:50	1,447	434.29	330,047	103,080
*Rosa chinensis*	Plant	0:33:21	53	515.12	517,448	136,774
*Selaginella moellendorffii*	Plant	0:08:31	757	212.32	66,930	21,034
*Sesamum indicum*	Plant	7:21:02	16,236	275.06	212,006	54,890
*Sorghum bicolor*	Plant	0:37:35	869	709.34	191,296	77,723
*Vitis vinifera*	Plant	2:30:58	1,907	486.2	442,690	102,253
*Zea mays*	Plant	1:24:40	687	2,182.79	361,036	109,725
*Homo sapiens*	Human	13:18:20	705	2,866.14	3,505,337	641,682
*Bos taurus*	Mammal	14:29:14	1,957	2,711.21	1,811,926	546,393
*Equus caballus*	Mammal	16:33:10	4,701	2,474.92	1,063,403	398,350
*Mus musculus*	Mammal	6:19:22	61	2,728.22	3,786,732	1,050,910
*Ovis aries*	Mammal	2:47:21	142	2,831.43	1,725,489	546,381
*Danio rerio*	Fish	14:07:36	1,923	1,679.20	2,714,042	493,206
*Fundulus heteroclitus*	Fish	4:23:42	1,031	1,203.51	1,396,976	417,662
*Anas platyrhynchos*	Bird	3:19:55	756	1,186.37	1,317,493	452,307
*Coturnix japonica*	Bird	3:12:31	2012	912.89	601,196	233,157
*Gallus gallus*	Bird	1:04:34	214	1,053.33	739,875	279,302
*Anolis carolinensis*	Reptile	23:50:10	6,457	1,799.14	1,253,882	383,060
*Acropora digitifera*	Cnidaria	0:27:38	2,421	431.66	89,034	46,032
*Bombyx mori*	Insect	0:30:10	697	452.05	245,570	60,888
*Drosophila melanogaster*	Insect	0:21:57	1,870	143.73	104,928	51,428
*Caenorhabditis elegans*	Worm	0:02:18	7	100.29	27,814	13,517
*Amphimedon queenslandica*	Sponge	2:04:52	13,133	165.983	102,435	38,340
*Emiliania huxleyi*	Plankton	3:31:32	7,795	167.68	181,243	23,032
*Tetrahymena thermophila*	Ciliate	0:10:10	1,158	103.01	33,530	3,343
*Xenopus tropicalis*	Amphibian	1:11:24	167	1,451.30	810,086	248,921
*Dictyostelium discoideum*	Amoeba	0:03:10	41	34.2	375,451	16,174
*Astrephomene gubernaculifera*	Algae	0:03:38	207	103.86	62,607	35,051
*Chlamydomonas reinhardtii*	Algae	0:05:03	53	111.1	144,980	60,669
*Saccharomyces cerevisiae*	Yeast	0:00:31	17	12.16	4,792	1,994
*Escherichia coli*	Bacteria	0:00:19	1	4.64	7	7

Srivastava et al. (2019) investigated the pattern of SSRs in genomic features and reported that about 60-80% of SSRs in land plants are located in intergenic regions, confirming the report of Lawson and Zhang (2006) in *Arabidopsis thaliana*. To compare this finding with the MegaSSR results, the *Arabidopsis thaliana* genome (5 chromosomes) was used with the default parameters (Implementation section), except that the compound SSR motifs were set to zero. MegaSSR identified a total of 56,071 SSR motifs, of which 35,156 (62.7%) were found in intergenic regions and 20,915 (37.3%) in genic regions. This result is consistent with previous findings by Srivastava et al. (2019) and Lawson and Zhang (2006).

### 3.3 Case study 2: Detection of SSRs at the transcriptome level

A total of 113 plant transcriptome sequences with a total size of 4,141.64 Mb, corresponding to 9,266,623 sequences, were retrieved from CyVerse Data Commons (One Thousand Plant Transcriptomes Initiative, 2019). These sequences were used to verify the performance of MegaSSR at the transcriptome level. Accordingly, using the default parameters (implementation subsection), with the exception of mononucleotides, which were excluded from the analysis, a total of 1,909,098 SSR motifs and 245,937 EST-SSR primers were detected. Using 56 CPUs, the average execution time was 4 minutes. **Supplementary Table 3** lists for each transcriptome the download link, the sequence size, the total number of sequences examined, the total number of SSRs identified, the number of SSR-containing sequences, the number of SSRs present in the compound, the total number of EST-SSR primers, the abundance of SSR classes, and links to the results.

### 3.4 Technical validation

To confirm the quality of the SSRs identified by MegaSSR, previously published data from the date palm (Mokhtar et al., 2016) and maize (Qu and Liu, 2013) were used for comparison. These data were selected because they broadly cover the genome and their accuracy was assessed by *in vitro* validation. The genome sequence of *Phoenix dactylifera* (Al-Dous et al., 2011) was downloaded from https://qatar-weill.cornell.edu/research/research-highlights/date-palm-research-program/date-palm-draft-sequence and analyzed using MegaSSR. The genome sequence contains 57,277 scaffolds with a size of approximately 381 Mbp, which were analyzed by Mokhtar et al. (2016) and therefore used for comparison with MegaSSR. The parameters used were mononucleotide ≥10 units, dinucleotide ≥6 units, and ≥5 units for all higher order motifs including trinucleotide, tetranucleotide, pentanucleotide, and hexanucleotide. For compound SSR motifs, the maximum difference between the two motifs was 100 bp. As a result, a total of 172,075 SSRs were identified, including 108,096 mono-, 48,156 di-, 11,841 tri-, 3,329 tetra-, 474 penta-, and 179 hexa-nucleotides. The current results are consistent with a previous study by Mokhtar et al. (2016) in which a total of 172,075 SSRs were identified using the MISA tool. A total of 172,075 SSR sequences reported by Mokhtar et al. (2016) were extracted from the genome sequences and used for comparison with the MegaSSR results. To compare these SSR repeats, SSRs and their flanking regions (200 bp) were extracted from the genome sequence and examined using the OrthoFinder tool (Emms and Kelly, 2019). OrthoFinder grouped the 172,075 SSR sequences (previous study) into 144,010 ortho groups and mapped them to the 172,075 SSR sequences in the MegaSSR results (**Supplementary Table 4**). This is due to the fact that SSRs can be multi-allelic, meaning that more than one sequence can be assigned to a group. The results showed that all SSR sequences reported by Mokhtar et al. (2016) matched the MegaSSR results.

Additionally, the whole genome of maize B73 (version ZmB73 RefGenV2) was downloaded from https://download.maizegdb.org/B73_RefGen_v2/ RefGen v2 and analyzed using MegaSSR. The ZmB73 RefGenV2 genome contains 10 chromosomes, mitochondria, chloroplast, and unmapped sequences. Only the 10 chromosomes (2.06 Gbp) were analyzed by Qu and Liu (2013), and therefore they were used for comparison with MegaSSR, which identified a total of 179,688 SSRs, including 47,830 mono-, 43,162 di-, 35,635 tri-, 2,616 tetra-, 800 penta-, 449 hexa-nucleotides, and 49,196 compound SSRs. Two studies by Qu and Liu (2013) and by (Pandey et al. 2018) reported a total of 179,681 SSRs using the MISA (Thiel et al. 2003) and WGSSAT (Pandey et al. 2018) tools, while MegaSSR reported 7 additional SSRs. A total of 82,694 SSRs with unique flanking sequences reported by Qu and Liu (2013) were extracted from the genome sequences and used for comparison with the MegaSSR results. To compare these SSR repeats, the SSRs and their flanking regions (200 bp) were extracted from the genome sequence and examined using the OrthoFinder tool (Emms and Kelly, 2019). OrthoFinder grouped the 82,694 SSR sequences (previous study) into 80,862 ortho groups and mapped them to the 84,239 SSR sequences in the MegaSSR results (**Supplementary Table 5**). The results showed that all 82,694 SSR sequences reported by Qu and Liu matched the MegaSSR results.

### 3.5 Comparison with other SSR web servers and tools

Existing SSR analysis tools provide useful data on SSR in both genomes and transcriptomes level. However, some of them have limitations, such as the ability to localize SSR primers or to detect genic and non-genic SSR. Some tools limit the size of the input sequence, and others are only available as standalone tools. Powerful tools are available as web servers, but they lack important features, limiting their usability (**Table 2**). PolyMorphPredict (Das et al., 2019), for example, is a web server that can analyze both DNA and EST sequences. It has a size limitation and does not classify SSRs based on gene proximity (genic and

**Table 2:** Comparison of some features provided by current SSR web servers.

Web server	SSRrepeats	Primerdesign	Classification into genic/nongenic	Stand-alone	Availability
MegaSSR	✓	✓	✓	✓	https://bioinformatics.um6p.ma/MegaSSR
PolyMorphPredict	✓	✓	X	X	http://webtom.cabgrid.res.in/polypred
SAT	-	-	-	✓	No longer available
ImtRDB	✓	✓	X	X	http://bioinfodbs.kantiana.ru/ImtRDB
AARTI	–	–	–	X	No longer available
ESMP	–	–	–	X	No longer available
ESAP Plus	✓	✓	X	X	http://gbp.kku.ac.th/esap_plus
WebSat	–	–	–	X	No longer available
SSRPrimer	–	–	–	X	No longer available
IMEx	–	–	–	X	No longer available
MICAS	✓	✓	X	X	http://www.mcr.org.in/micas

"✓" refer to the feature is found, "X" refers to the feature is missing, and "-" refer to the feature not checked because the web server is no longer available.

non-genic). ESAP Plus (Ponyared et al., 2016) is another web server for SSR analysis. However it requires registration and is designed for EST sequences only. MICAS (Sreenu et al., 2003) is a web server limited to SSR analysis of prokaryotic and viral genome sequences and cannot process eukaryotic genomes.

Other SSR web servers exist in the literature and are no longer available, such as the AutomAted RepeaT Identifier (AARTI, https://lms.snu.edu.in/aarti) Kumar et al. (2022), EST-SSR Marker Pipeline (ESMP, https://bioinfo.aau.ac.in/ESMP) Sarmah et al. (2012), WebSat (https://purl.oclc.org/NET/websat) Martins et al. (2009), SSRPrimer (http://bioinformatics.pbcbasc.latrobe.edu.au/ssrdiscovery.html) (Jewell et al., 2006), and Imperfect Microsatellite Extractor (IMEx, http://www.cdfd.org.in/imex) (Mudunuri and Nagarajaram, 2007). SSR Analysis Tool (SAT, http://sat.cirad.fr/sat) (Dereeper et al., 2007) is a web server and standalone application for SSR search and primer design. However, the web server is no longer available and the standalone tool is available upon request. Some SSR databases, such as PolySSR (Tang et al., 2008), SSRome (Mokhtar and Atia, 2019), and ImtRDB (Shamanskiy et al., 2019) provide analysis capabilities for SSR data. PolySSR is a pipeline for EST-SSR analysis and includes EST-SSR primers for tomato, rice, Arabidopsis, potato, brassica, and chicken. It is available through https://www.bioinformatics.nl/tools/polyssr/ but is limited to the analysis of SSRs in the aforementioned six genomes. SSRome (http://mggm-lab.easyomics.org), on the other hand, is a dynamic database with pipelines for the analysis of SSRs in 6,533 organisms. However, SSRome only provides analysis of stored genomes and does not provide an option to upload and analyze new sequences. ImtRDB is another database and software designed for mitochondrial and chloroplastic SSRs and is not suitable for whole-genome or transcriptome detection and analysis of SSRs.

## 4 Conclusion

MegaSSR is a web-based server and a standalone for microsatellite investigation and analysis, and for the design of targeted SSR PCR-based primers at the whole genome and transcriptome level. This pipeline includes basic SSR mining methods such as SSR identification and primer design for basic methods. However, it also includes advanced methods such as classification of SSR motifs based on their proximity to genic and non-genic motifs. In addition to determining which SSR motifs occur only in genic or nongenic regions, we also classify the shared SSRs between the two regions. As a result, it provides active statistical visualization methods such as tables and graphs, as well as the ability to locate SSR motifs and designed primers at the genome level using the JBrowse tool. MegaSSR provides essential tools for genetic diversity research and marker design. MegaSSR can be used to find SSRs and design PCR primers that target flanking regions of SSRs. Users can screen and compare genic and non-genic regions based on their SSR repeat content. In addition, the PCR primers allow specific targeting of these regions. MegaSSR provides dynamic graphs that allow users to visualize the data and select PCR primers efficiently.

## Data availability statement

The original contributions presented in the study are included in the article/**Supplementary Material**. Further inquiries can be directed to the corresponding authors. MegaSSR web server is freely available at: https://bioinformatics.um6p.ma/MegaSSR and the standalone version at https://github.com/MoradMMokhtar/MegaSSR.

## Author contributions

Conceptualization: MM and AEA; Methodology: MM, AMA and AEA; Scripting: MM and AEA; Data curation: MM and AMA; Writing–original draft: MM, AMA and AEA. All authors reviewed the manuscript. All authors contributed to the article and approved the submitted version.
